# From precision to planetary health: the carbon cost of AI in multimodality cardiovascular imaging

**DOI:** 10.1093/ehjimp/qyag078

**Published:** 2026-04-28

**Authors:** Théo Pezel, Alexandre Unger, Julien Hudelo, Sofiane Sifaoui, Aicha Kante, Camille Gersdorff, Trecy Gonçalves, Solenn Toupin

**Affiliations:** MIRACL.ai laboratory, Multimodality Imaging for Research and Analysis Core Laboratory and Artificial Intelligence, University Hospital of Lariboisiere, Assistance Publique des Hôpitaux de Paris (AP-HP), Paris 75010, France; Université Paris Cité (UPC), Inserm MASCOT - UMRS 942, Paris 75010, France; Department of Cardiology, University Hospital of Lariboisiere, (Assistance Publique des Hôpitaux de Paris, AP-HP), Paris 75010, France; MIRACL.ai laboratory, Multimodality Imaging for Research and Analysis Core Laboratory and Artificial Intelligence, University Hospital of Lariboisiere, Assistance Publique des Hôpitaux de Paris (AP-HP), Paris 75010, France; Université Paris Cité (UPC), Inserm MASCOT - UMRS 942, Paris 75010, France; Department of Cardiology, University Hospital of Lariboisiere, (Assistance Publique des Hôpitaux de Paris, AP-HP), Paris 75010, France; Department of Cardiology, Hôpital universitaire de Bruxelles, Hôpital Erasme, Université libre de Bruxelles, Brussels, Belgium; MIRACL.ai laboratory, Multimodality Imaging for Research and Analysis Core Laboratory and Artificial Intelligence, University Hospital of Lariboisiere, Assistance Publique des Hôpitaux de Paris (AP-HP), Paris 75010, France; Université Paris Cité (UPC), Inserm MASCOT - UMRS 942, Paris 75010, France; Department of Cardiology, University Hospital of Lariboisiere, (Assistance Publique des Hôpitaux de Paris, AP-HP), Paris 75010, France; Department of Cardiology, Amiens University Hospital, UR UPJV 7517, Jules Verne University of Picardie, Amiens, France; MIRACL.ai laboratory, Multimodality Imaging for Research and Analysis Core Laboratory and Artificial Intelligence, University Hospital of Lariboisiere, Assistance Publique des Hôpitaux de Paris (AP-HP), Paris 75010, France; Université Paris Cité (UPC), Inserm MASCOT - UMRS 942, Paris 75010, France; Department of Cardiology, University Hospital of Lariboisiere, (Assistance Publique des Hôpitaux de Paris, AP-HP), Paris 75010, France; MIRACL.ai laboratory, Multimodality Imaging for Research and Analysis Core Laboratory and Artificial Intelligence, University Hospital of Lariboisiere, Assistance Publique des Hôpitaux de Paris (AP-HP), Paris 75010, France; Université Paris Cité (UPC), Inserm MASCOT - UMRS 942, Paris 75010, France; Department of Internal Medecine, University Hospital of Lariboisiere, (Assistance Publique des Hôpitaux de Paris, AP-HP), Paris 75010, France; MIRACL.ai laboratory, Multimodality Imaging for Research and Analysis Core Laboratory and Artificial Intelligence, University Hospital of Lariboisiere, Assistance Publique des Hôpitaux de Paris (AP-HP), Paris 75010, France; Université Paris Cité (UPC), Inserm MASCOT - UMRS 942, Paris 75010, France; Department of Cardiology, University Hospital of Lariboisiere, (Assistance Publique des Hôpitaux de Paris, AP-HP), Paris 75010, France; MIRACL.ai laboratory, Multimodality Imaging for Research and Analysis Core Laboratory and Artificial Intelligence, University Hospital of Lariboisiere, Assistance Publique des Hôpitaux de Paris (AP-HP), Paris 75010, France; Université Paris Cité (UPC), Inserm MASCOT - UMRS 942, Paris 75010, France; Department of Cardiology, University Hospital of Lariboisiere, (Assistance Publique des Hôpitaux de Paris, AP-HP), Paris 75010, France; MIRACL.ai laboratory, Multimodality Imaging for Research and Analysis Core Laboratory and Artificial Intelligence, University Hospital of Lariboisiere, Assistance Publique des Hôpitaux de Paris (AP-HP), Paris 75010, France; Université Paris Cité (UPC), Inserm MASCOT - UMRS 942, Paris 75010, France; Department of Cardiology, University Hospital of Lariboisiere, (Assistance Publique des Hôpitaux de Paris, AP-HP), Paris 75010, France

## Abstract

**Graphical Abstract:**
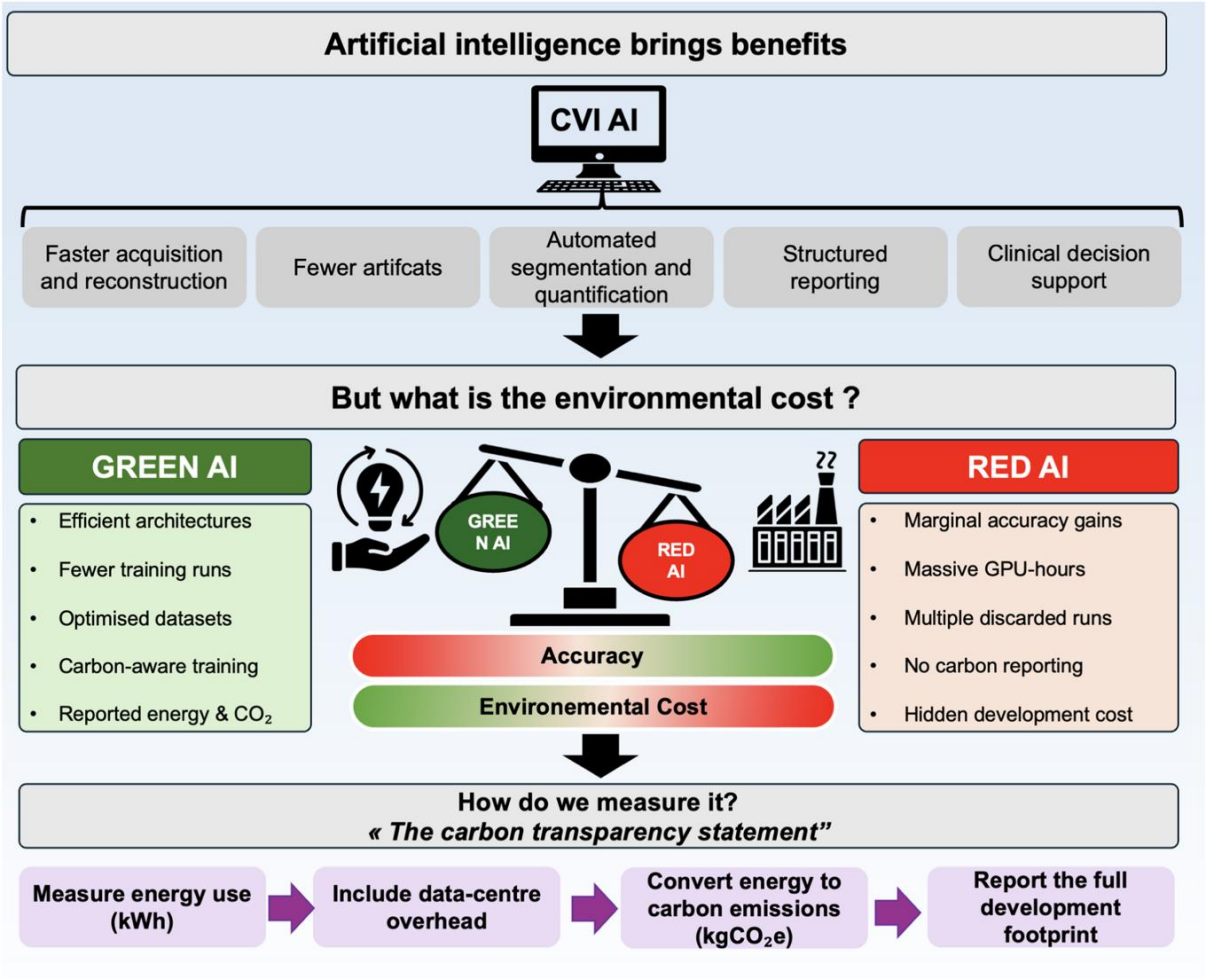
Balancing clinical benefits and environmental cost of AI in cardiovascular imaging. Top panel. AI provides major benefits in cardiovascular imaging, including faster acquisition and reconstruction, fewer artefacts, automated segmentation and quantification, structured reporting, and clinical decision support. Middle panel. Comparison between Green AI and Red AI paradigms. While clinical performance may be similar, Green AI prioritizes efficiency and transparency, whereas Red AI is associated with disproportionate computational demand and unreported environmental costs. Bottom panel. Proposed framework for a carbon transparency statement, based on measuring energy consumption, accounting for data-centre overhead, converting energy use to carbon emissions, and reporting the full AI development footprint. AI, artificial intelligence; CVI, cardiovascular imaging; CO_2_, carbon dioxide; GPU, graphics processing unit; kgCO_2_e, kilograms of carbon dioxide equivalent.

Balancing clinical benefits and environmental cost of AI in cardiovascular imaging. Top panel. AI provides major benefits in cardiovascular imaging, including faster acquisition and reconstruction, fewer artefacts, automated segmentation and quantification, structured reporting, and clinical decision support. Middle panel. Comparison between Green AI and Red AI paradigms. While clinical performance may be similar, Green AI prioritizes efficiency and transparency, whereas Red AI is associated with disproportionate computational demand and unreported environmental costs. Bottom panel. Proposed framework for a carbon transparency statement, based on measuring energy consumption, accounting for data-centre overhead, converting energy use to carbon emissions, and reporting the full AI development footprint. AI, artificial intelligence; CVI, cardiovascular imaging; CO_2_, carbon dioxide; GPU, graphics processing unit; kgCO_2_e, kilograms of carbon dioxide equivalent.

Artificial intelligence (AI) has transitioned from a supporting adjunct to a defining pillar of cardiovascular imaging.^1^ In echocardiography, cardiac computed tomography (CT), cardiac magnetic resonance imaging (MRI), and nuclear cardiology, AI systems now promise faster acquisition and reconstruction, fewer artefacts, automated segmentation and quantification, structured reporting, and even ‘one click’ clinical decision support. This trajectory is not only technological; it has a real cultural impact. We increasingly equate progress with larger datasets, deeper networks, and faster graphics processing units (GPUs).^2^ However, in a health sector that is already expected to decarbonize, there is an uncomfortable question we have largely avoided: what is the ecological price of our AI-enabled ‘efficiency’, and who is accountable for paying it?

That question is not philosophical but has real implications for our patients. Health care is a material emitter, responsible for a meaningful proportion of global greenhouse gas emissions.^3^ Exposure to ambient air pollution has been associated with an estimated reduction in life expectancy of nearly 3 years and is thought to account for ∼8.8 million excess deaths worldwide each year.^4^ For that reason, the European Society of Cardiology (ESC) has provided guidelines on cardiovascular disease prevention in clinical practice, identifying air pollution as a major threat to human health and a key contributor to climate change, primarily driven by fossil fuel use and increasing carbon dioxide (CO_2_) emissions.^5^

In a recent EACVI survey including 218 cardiovascular imaging professionals from 41 countries, only 11% reported having received formal education on climate change or sustainable healthcare, although 41% had already encountered patients whose cardiovascular health was potentially affected by climate-related factors.^6^ While 51% believed that healthcare professionals should play a major role in addressing the health consequences of climate change, 59% reported that the environmental impact of imaging examinations had little or no influence on their clinical decision-making. Most respondents recognized human activity as the main driver of climate change (80%) and identified energy consumption as the primary determinant of imaging-related environmental impact (73%). However, only 20% were aware of the contribution of the health sector to global greenhouse gas emissions. Radiology and cardiovascular imaging are energy-intensive components of that footprint, and AI is poised to add a new layer of computational demand on top of already carbon-hungry modalities.^7^ The concern is not that AI should be abandoned; rather, that we should stop treating its environmental externalities as irrelevant ‘background noise’ and start reporting them with the same rigour we apply to safety and performance.

## AI is now embedded across the imaging pathway

AI’s integration in the field of cardiovascular imaging and research is rapidly expanding. At acquisition, AI-driven denoising, motion correction, and accelerated reconstruction shorten scan times and enhance image quality, reducing repeats and increasing throughput. In post-processing, deep learning enables automated segmentation and quantification of volumes, function, perfusion, scar, and strain, increasingly framed as productivity tools. At the reporting level, natural language generation and structured templates seek to standardize outputs, limit variability, and shorten reporting delays. In research, the appetite is even larger. Supervised models seek to predict clinical outcomes from multimodal imaging fused with clinical, biomarker, and omics data.^8^ Unsupervised learning (clustering) promises to uncover cardiovascular phenogroups and mechanistic hypotheses, with the implicit ambition of identifying actionable therapeutic targets. This is the seductive narrative: AI does not only automate, it discovers (*Graphical Abstract*).

## The uncomfortable asymmetry: training dominates, and “Red AI’ is rewarded

The machine learning community has been warning for years that the pursuit of marginal accuracy gains can drive disproportionate computational cost.^9^ The ‘Green AI’ argument is blunt: efficiency should be a first-class scientific metric, not an afterthought.^10^ Earlier work quantified the financial and environmental costs of training large models, catalyzing debate about whether the field’s incentives quietly reward energy-intensive experimentation. More recent work has pushed for systematic, standardized reporting of energy and carbon footprints as a basic component of scientific transparency.^9^

Medicine, however, has imported the accuracy-first culture of computer science without importing the corresponding accountability norms. In cardiovascular imaging, we routinely read papers presenting ‘state-of-the-art’ performance without any indication of how many GPU hours were consumed, how many experiments were run and discarded, or what the associated greenhouse gas emissions were.^2^ We also rarely distinguish between the carbon cost of a single final training run and the much higher cumulative cost of development: data curation, preprocessing, repeated training due to hyperparameter tuning, ablation studies, debugging cycles, and failed experiments that never make it into the Methods sections of published articles.^2,9^

The asymmetry matters because training is typically far more energy-intensive than evaluation or inference. Without measurement, we will not know whether our ‘incremental AUC gain’ comes at an ecologically disproportionate training cost.^2^ These considerations, therefore, motivate a paradigm shift towards Frugal AI, i.e., AI solutions designed to attain high performance with minimal resources. This can be achieved at the hardware level by reducing compute and memory consumption, at the algorithmic level by using more efficient model architectures and training strategies, but also at the data level by reducing dependence on large, annotated datasets.

## A pragmatic approach to measuring the carbon footprint of AI training in imaging

The good news is that measuring the carbon footprint of AI is feasible.^2^ We do not need perfect estimates to begin; we need consistent, transparent, reproducible ones.

A practical framework can be built from four components^11^:

1. Measure energy consumption (kWh) for the computation you actually control: use direct instrumentation when possible (server power meters, scheduler-reported energy, on-board sensors) and software trackers when not. Tools exist to estimate and log emissions from AI computing workloads, including CodeCarbon (https://codecarbon.io) and related approaches explicitly designed for machine learning experiments.
2. Account for data-centre overhead: if you train on a cluster, include the power usage effectiveness (PUE) or an institutional estimate: Total energy (kWh) = Information technology energy (kWh) × PUE. If PUE is unknown, report assumptions and perform sensitivity analyses.
3. Convert energy to carbon emission (kgCO_2_e): multiply electricity consumed by a carbon intensity factor for the grid supplying the compute. This is precisely why location matters: the same training run in a low-carbon grid can have a very different footprint than in a fossil-heavy grid. The Greenhouse Gas Protocol distinguishes location-based and market-based accounting for purchased electricity; reporting both can improve transparency, particularly when renewable contracts are claimed, as follows:
  $$\mathrm{Emissions}\;(\mathrm{kgC}O_{2}e)=\mathrm{Total}\;\mathrm{energy}\;(\mathrm{kWh})\times \mathrm{Grid}\;\mathrm{intensity}\;(\mathrm{kgC}O_{2}e/\mathrm{kWh})$$
4. Report the full development budget, not only the final model, with at minimum: total GPU-hours, number of training runs, peak power draw, wall-clock duration, dataset size, and the emissions for the final selected model, plus an estimate of cumulative development emissions. The argument for systematic reporting has been made clearly: without standardized disclosure, we cannot compare methods fairly or incentivize efficiency.

This should be paired with a simple ‘carbon label’ for medical AI: emissions per training run, cumulative emissions for development, and emissions per 1000 inferences (or per patient) in the intended deployment setting.^11^

## Why journals and reviewers should demand ‘carbon transparency statements’

Medical AI is now mature enough that transparency expectations should rise. We already require trial registration, data availability statements, and, in many contexts, adherence to reporting guidance (including AI-specific extensions). A carbon transparency statement is not bureaucratic ornamentation; it is a scientific context. Two models with identical discrimination and calibration are not equivalent if one requires 50 times more energy to develop.^11^ The latter may be less reproducible, less equitable (only a few centres can afford to replicate it), and less defensible in an era where health systems are committing to a net zero balance between greenhouse gas emission and removal, with no impact on climate change. The National Health Service in the UK, for example, has made explicit net-zero commitments and tracks progress over time.

Making carbon visible will also trigger healthier controversy. It will force us to ask: are we practicing ‘precision medicine’, or are we engaging in ‘prestige computing’? Do we pursue a 0.01 improvement in *C*-statistic because it changes care, or because it wins benchmarks? In cardiovascular imaging, where clinical pathways already face scrutiny for appropriateness and cost, ignoring environmental cost is becoming increasingly indefensible.^2,11^

Evaluating the carbon footprint of AI may also open the door to assessing its positive impacts beyond conventional clinical performance metrics. By accelerating image acquisition, shortening sequences, reducing repeat scans, optimizing scanner utilization, and promoting more appropriate imaging across care pathways, AI can act as a lever for environmental efficiency.^12^ These technical and organizational gains have the potential to lower energy consumption and downstream emissions in routine practice, suggesting that AI evaluation frameworks should also acknowledge their capacity to contribute to a net reduction in the carbon footprint of cardiovascular imaging workflows.

## Conclusion

Cardiovascular imaging has entered an AI era at the exact moment health systems must decarbonize. We cannot keep publishing machine learning and deep learning studies as if computation were ecologically free. The field should move rapidly towards routine carbon reporting for AI development and deployment, ideally in a harmonized format that enables comparison across studies and modalities. The tools and frameworks exist; the barrier is cultural, not technical. If we succeed, we will not slow innovation; we will improve it. We will incentivize efficiency, reproducibility, and equity. We will reduce the temptation to equate ‘bigger’ with ‘better’. We will align cardiovascular imaging research with a broader ethical mandate: protecting health includes protecting the planetary conditions that make health possible.
